# Baseline ^18^F-FDG PET/CT parameters in predicting the efficacy of immunotherapy in non-small cell lung cancer

**DOI:** 10.3389/fmed.2025.1477275

**Published:** 2025-01-31

**Authors:** Lu Zheng, Yanzhu Bian, Yujing Hu, Congna Tian, Xinchao Zhang, Shuheng Li, Xin Yang, Yanan Qin

**Affiliations:** ^1^Department of Nuclear Medicine, Hebei General Hospital, Shijiazhuang, China; ^2^Hebei Provincial Key Laboratory of Cerebral Networks and Cognitive Disorders, Shijiazhuang, China; ^3^Department of Nuclear Medicine, Affiliated Hospital of Hebei University, Baoding, China

**Keywords:** PET/CT, SUV, non-small cell lung cancer, immune checkpoint inhibitors, prognosis

## Abstract

**Objective:**

To analyse positron emission tomography/ computed tomography (PET/CT) imaging and clinical data from patients with non-small cell lung cancer (NSCLC), to identify characteristics of survival beneficiaries of immune checkpoint inhibitors (ICIs) treatment and to establish a survival prediction model.

**Methods:**

A retrospective analysis was conducted on PET/CT imaging and clinical parameters of 155 NSCLC patients who underwent baseline PET/CT examination at the Department of Nuclear Medicine, Hebei General Hospital. The Kaplan–Meier curve was employed to compare progression-free survival (PFS) and overall survival (OS) between the ICIs and non-ICIs group and to assess the impact of variables on PFS and OS in the ICIs group. Multivariate Cox proportional hazards regression analysis was conducted with parameters significantly associated with survival in univariate analysis.

**Results:**

Significant differences were observed in PFS (*χ^2^* = 11.910, *p* = 0.0006) and OS (*χ^2^* = 8.343, *p* = 0.0039). Independent predictors of PFS in the ICIs group included smoking history[hazard ratio (HR) = 2.522, 95% confidence interval (CI): 1.044 ~ 6.091, *p* = 0.0398], SUVmax of the primary lesion(HR = 0.2376, 95%CI: 0.1018 ~ 0.5548, *p* = 0.0009), MTVp (HR = 0.0755, 95%CI: 0.0284 ~ 0.2003, *p* < 0.001), and TLGp (HR = 0.1820, 95%CI: 0.0754 ~ 0.4395, *p* = 0.0002). These were also independent predictors of OS in the ICIs group[HR(95%CI) were 2.729 (1.125 ~ 6.619), 0.2636 (0.1143 ~ 0.6079), 0.0715 (0.0268 ~ 0.1907), 0.2102 (0.0885 ~ 0.4992), both *p* < 0.05)]. Age was an additional independent predictor of OS (HR = 0.4140, 95%CI: 0.1748 ~ 0.9801, *p* = 0.0449).

**Conclusion:**

Smoking history, primary lesion SUVmax, MTVp, and TLGp were independent predictors of PFS, whilst age, smoking history, SUVmax, MTVp, and TLGp were independent predictors of OS in the ICIs group. Patients without a history of smoking and with SUVmax ≤19.2, MTVp ≤20.745cm^3^, TLGp ≤158.62 g, and age ≤ 60 years benefited more from ICI treatment.

## 1 Introduction

Lung cancer is a primary malignancy originating from the trachea, bronchial mucosa, or glands, and can be classified into small cell lung cancer (SCLC) and non-small cell lung cancer (NSCLC) based on histological heterogeneity. NSCLC accounts for approximately 80–85% of all lung cancers, with a 5-year survival rate of only 10–16% for patients with advanced NSCLC (1, 2). According to the China’s latest cancer survey in 2022, lung cancer ranks first in incidence and mortality (3). Advances in medical treatment have led to an increasing number of immune checkpoint inhibitors (ICIs) being approved for lung cancer in China, and the application of immunotherapy in the treatment of NSCLC has gradually become more widespread. Preliminary studies (4, 5) have suggested that ^18^F-fluorodeoxyglucose (^18^F-FDG) uptake is associated with the efficacy of immunotherapy, with higher maximum standard uptake value (SUVmax) correlating with better treatment outcomes. However, higher metabolic tumour volume (MTV) and total lesion glycolysis (TLG) are associated with a higher non-response rate and worse prognosis in NSCLC patients treated with ICIs. The objective response rate to ICIs is approximately 20% with 10% of patients experiencing serious immune-related adverse events (irAEs) (6). This study aims to analyse positron emission tomography/ computed tomography (PET/CT) imaging and clinical data of NSCLC patients treated with and without ICIs, comparing differences in progression-free survival (PFS) and overall survival (OS), and identifying characteristics of patients who benefit from ICIs. A survival prediction model based on ^18^F-FDG PET/CT parameters is developed to aid in the screening of NSCLC patients for immunotherapy.

## 2 Materials and methods

### 2.1 Patients

Clinical and imaging data of NSCLC patients who underwent baseline ^18^F-FDG PET/CT were retrospectively reviewed at Hebei General Hospital from January 2016 to April 2023. Inclusion criteria were: patients with pathologically confirmed NSCLC, no history of other malignancies or conditions affecting imaging agent uptake, ^18^F-FDG PET/CT performed within 1 week before treatment, and complete medical records. Exclusion criteria included patients unable to remain supine for an extended period during the examination and those who were lactating or pregnant.

A total of 174 patients’ baseline clinical information was collected and 155 patients were enrolled in the study after screening. This cohort included 67 patients treated with ICIs and 88 patients who were not treated with ICIs. Collected clinical data comprised age, gender, height, weight, body mass index (BMI), smoking history and status, drinking habits, family history of cancer, pathological type, Ki-67%, stage, and various tumour markers [carcinoembryonic antigen(CEA), squamous cell carcinoma antigen(SCC), Cytokeratin 19 fragment(CYFRA21-1), neuron specific enolase(NSE)], as well as lactate dehydrogenase(LDH), white blood cells(WBC) count, neutrophilic granulocyte(NE) count, erythrocyte sedimentation rate(ESR), and C-reactive protein (CRP).

### 2.2 ^18^F-FDG PET/CT examination

Before the examination, patients fasted for at least 6 h and had fasting blood glucose levels ≤11.1 mmol/L. Body mass and height were measured. The ^18^F-FDG was provided by Hebei Andike Positron Technology Co., with a radiochemical purity of ≥95%. It was administered intravenously at a dose of 3.7–5.5 MBq/kg (0.10–0.15 mCi/kg) approximately 60 min before the PET/CT examination. Scanning was performed using the GE Discovery Elite PET/CT device, covering from the base of the skull to the middle and upper third of the thigh. CT parameters were set at 120 kV, 100 mA tube current, and 3.3 mm layer thickness. PET parameters included a 3D-TOF method for collecting PET images over 6–7 beds, with a 2-min acquisition time per bed. Images were reconstructed using full energy X-ray attenuation correction and ordered subset expectation maximisation algorithm, with a layer thickness of 3.3 mm. PET, CT and PET/CT fusion images were obtained, along with chest CT images reconstructed using a filtered back projection method with a 5 mm layer thickness and 1.25 mm thin-layer images.

### 2.3 Image analysis

PET/CT images were evaluated by two nuclear medicine physicians with advanced qualifications. Regions of interest (ROI) for each hypermetabolic lesion were identified on the PET/CT images, and the SUVmax was quantified. The mean standardized uptake value (SUVmean), minimum standardized uptake value (SUVmin), MTV of primary (MTVp) and TLG of primary (TLGp) were automatically extracted using a threshold of 40% of each SUVmax. The whole-body MTV(MTVwb) and whole-body TLG(TLGwb) for all lesions in each patient were calculated after determining the SUVmean and MTV for each ROI of all metastases. A circular ROI with a diameter of 1.0 cm (one in the left lobe and two in the right lobe) was delineated at normal metabolic sites in the liver, and the average SUVmax at these sites was used to calculate the tumor/liver ratio (TLR) of SUVmax (7). Lymph node and distant metastases in the PET/CT images were recorded. The characteristics of the primary lesion, including location, density, lobulation, burr, calcification, cancerous lymphangitis, and pleural effusion, were noted from the thin-slice CT images of the chest. The CT values of the primary lesion and the lengths of three radial lines were measured, with the maximum radial line length recorded as the maximum radial value.

### 2.4 Follow-up

Patients’ outcome information was obtained from electronic medical records, imaging reports, or telephone follow-ups until November 30, 2023. The follow-up duration ranged from 7 to 89 months, with a median of 26 months. Outcomes were classified according to response evaluation criteria in solid tumor (RECIST) criteria as complete response (CR), partial response (PR), progressive disease (PD), or stable disease (SD) (8). PFS and OS were recorded. PFS was defined as the time from the pathological diagnosis of NSCLC to disease progression, death from any cause, or the follow-up cutoff time. OS was defined as the time from pathological diagnosis to death from any cause or the follow-up cutoff time (9).

### 2.5 Statistical analysis

SPSS 25.0 was employed for data analysis. The Shapiro–Wilk test was used to assess normality, with normally distributed measurement data expressed as mean ± standard deviation (±SD) and non-normally distributed data presented as interquartile range (IQR). Classification variables were described as frequency (percentage). The Kaplan–Meier curve was used to compare PFS and OS between the ICIs and non-ICIs groups. Continuous variables in the ICIs group were dichotomised based on the optimal cut-off value from the receiver-operating characteristics (ROC) curve. The Kaplan–Meier curve assessed factors influencing PFS and OS in the ICIs group, and the log-rank test was used to analyse differences between groups. GraphPad Prism 9 was used to plot the curves. Statistically significant parameters identified in univariate analysis were included in the Cox proportional hazards regression model for multivariate analysis to determine independent predictors of PFS and OS. The ROC curve was used to validate the diagnostic efficacy of the risk prediction model. *p* < 0.05 was considered statistically significant.

## 3 Results

### 3.1 Basic information

#### 3.1.1 Clinical data

Clinical data for the two groups are presented in Table 1. Amongst the 155 NSCLC, there are 67 cases in ICIs group with median age 65.0 (59.0, 70.0) years. The non-ICIs group had 88 cases with a median age of 67.0 (57.0, 74.8) years. There were more males than females in both groups (60 vs. 7.55 vs. 33). The most common pathologic types in the ICIs and non-ICIs groups were squamous carcinoma in 38 cases (56.7%) and adenocarcinoma in 63 cases (71.6%), respectively. There were no CR cases in both groups after follow-up.There were 8 (11.9%) cases of PR, 34 (50.7%) cases of SD, 25 (37.3%) cases of PD, 23 (34.3%) cases of death in the ICIs group. In the non-ICIs group, there were 1 (1.1%) PR, 28 (31.8%) SD, 59 (67.0%) PD, 58 (65.9%) deaths. TNM staging was determined according to the 8th edition of the lung cancer criteria (10).

**Table 1:** Clinical data of NSCLC patients in the ICIs and non-ICIS groups.

Clinical data	ICIs group	Non-ICIs group
Age/years	65.0 (59.0, 70.0)	67.0(57.0, 74.8)
Sex		
Male	60 (89.6%)	55 (62.5%)
Female	7 (10.4%)	33 (37.5%)
Height/cm	170.0 (168.0, 175.0)	168.0 (160.0, 172.0)
Body mess/kg	70.0 (62.5.0, 75.0)	65.5 (60.0, 75.0)
BMI/kg·m^−2^	24.22 (21.97, 25.95)	24.08 (21.69, 26.08)
Smoking history	31 (46.3%)	25 (28.4%)
Smoking status/pack-years	24 (0.40)	20 (0.30)
Drinking	8 (11.9%)	14 (15.9%)
Family history of cancer	14 (20.9%)	17 (19.3%)
Pathological type		
Adenocarcinoma	26 (38.8%)	63 (71.6%)
Squamous carcinoma	38 (56.7%)	17 (19.3%)
Adenosquamous carcinoma	1 (1.5%)	5 (5.7%)
Large cell carcinoma	1 (1.5%)	1 (1.1%)
No specific type	1 (1.5%)	0
Alveolar cell carcinoma	0	1 (1.1%)
Pulmonary blastoma	0	1 (1.1%)
Ki-67%	40.0 (30.0, 60.0)	30.0 (20.0, 55.0)
CEA/ng·ml^−1^	4.88 (2.62, 7.63)	12.26 (3.81, 49.19)
SCC/ng·ml^−1^	1.87 (1.26, 3.52)	1.52 (1.07, 2.21)
NSE/ng·ml^−1^	13.10 (10.04, 17.83)	14.08 (11.56, 17.60)
CYFRA21-1/ng·ml^−1^	11.10 (4.18, 18.20)	4.84 (3.43, 8.83)
LDH/U·L^−1^	175.70 (151.83, 208.03)	189.10 (165.68, 225.78)
WBC/L^−1^	7.18 (5.48, 8.73)	7.00 (5.90, 9.56)
NE/L^−1^	5.18 (3.78, 7.99)	4.90 (3.74, 7.00)
ESR/mm·h^−1^	14.00 (6.50, 37.00)	17.00 (10.00, 28.00)
CRP/mg·L^−1^	12.11 (3.19, 73.09)	12.69 (1.21, 55.78)
TNM stage		
I	1 (1.5%)	3 (3.4%)
II	2 (3.0%)	1 (1.1%)
III	21 (31.3%)	27 (30.7%)
IV	43 (64.2%)	57 (64.8%)
Follow-up		
CR	0	0
PR	8 (11.9%)	1 (1.1%)
SD	34 (50.7%)	28 (31.8%)
PD	25 (37.3%)	59 (67.0%)
Number of deaths	23 (34.3%)	58 (65.9%)
PFS/month	19.5 (10.5, 29.0)	15.0 (7.0, 28.5)
	(1.0 ~ 73.0)	(1.0 ~ 73.0)
OS/month	22.0 (12.0, 36.8)	20.0 (11.0, 36.0)
	(4.0 ~ 81.0)	(1.0 ~ 89.0)

CEA, carcinoembryonic antigen; SCC, squamous cell carcinoma antigen; NSE, neuron specific enolase; CYFRA21-1, Cytokeratin 19 fragment; LDH, lactate dehydrogenase; WBC, white blood cells; NE, neutrophilic granulocyte; ESR, erythrocyte sedimentation rate; CRP, C-reactive protein; CR, complete response; PR, partial response; PD, progressive disease; SD, stable disease; PFS, progression-free survival; OS, overall survival.

#### 3.1.2 PET/CT morphological characteristics and metabolic parameters

The primary lung lesions were predominantly located in the upper lobes, with 49 cases(31.6%, 49/155) in the right upper lobe and 42 cases(27.1%, 42/155) in the left upper lobe. Most lesions were peripheral (109 vs. 46). The morphology of the primary lesions varied, including lobulated, burr-like, lymphangitic spread of carcinoma, calcification, and pleural effusion. The density was predominantly homogeneous and solid. The average CT value of the primary lesions in the ICI group was 30.19 ± 9.85 HU, compared to 33.43 ± 10.47 HU in the non-ICI group. The largest diameter of the lesions was 39.0 mm (27.0, 62.0) in the ICI group and 35.0 mm (26.0, 51.0) in the non-ICI group (Table 2).

**Table 2:** PET/CT morphological characteristics of NSCLC patients in ICIs and non-ICIs group.

Morphological features	ICIs group	Non-ICIs group
Distribution of lung lobes		
Right lung		
Superior lobe	23	26
Middle lobe	2	5
Inferior lobe	14	18
Left lung		
Superior lobe	20	22
Inferior lobe	8	17
Intra-leaf distribution		
Central type	23	23
Peripheral type	44	65
Lobulation	49	70
Burr	56	78
Lymphangitic spread of carcinoma	13	11
Calcification	14	18
Pleural effusion	19	29
Density		
Solid	36	56
Solid with voids	10	15
Solid with necrosis	13	12
Solid with cavity and necrosis	4	3
Heterogeneous reality	4	2
Mean CT value /HU	30.19 ± 9.85	33.43 ± 10.47
Largest diameter /mm	39.0(27.0, 62.0)	35.0(26.0, 51.0)

The PET/CT manifestations of primary lung cancer were characterised by high metabolic activity. Lymph node metastasis was observed in 130 cases(83.9%, 130/155), whilst distant metastasis was noted in 103 cases (66.5%, 103/155). Metabolic parameters of the primary lesions, including SUVmax, SUVmean, SUVmin, TLR, MTVp, TLGp, as well as MTVwb and TLGwb of all lesions, were assessed in both the ICI and non-ICI groups (Table 3).

**Table 3:** Baseline PET/CT metabolic parameters of NSCLC patients in ICIs and non-ICIS groups.

Metabolic parameters	ICIs group	Non-ICIs group
SUVmax	14.5 (9.5, 21.9)	11.3 (7.9, 14.9)
SUVmean	8.2 (5.6, 11.9)	6.7 (4.6, 9.1)
SUVmin	4.9 (3.7, 7.0)	4.5 (3.2, 6.1)
TLR	5.17 (2.88, 6.72)	3.59 (2.61, 5.10)
MTVp/ cm^3^	12.08 (5.05, 32.08)	10.93 (5.11, 21.91)
TLGp/g	106.48 (32.97, 306.93)	78.76 (22.40, 191.24)
MTVwb/ cm^3^	70.72 (27.46, 120.60)	64.22 (28.10, 114.43)
TLGwb/g	378.11 (169.37, 923.52)	306.26 (167.28, 700.52)

SUVmax maximum standard uptake, SUVmean mean standardized uptake, SUVmin minimum standardized uptake, TLR tumor/liver ratio of SUVmax, MTVp metabolic tumour volume of primary, TLGp total lesion glycolysis of primary, MTVwb whole-body MTV, TLGwb whole-body TLG.

### 3.2 Kaplan–Meier survival curve analysis

**Figure 1:**
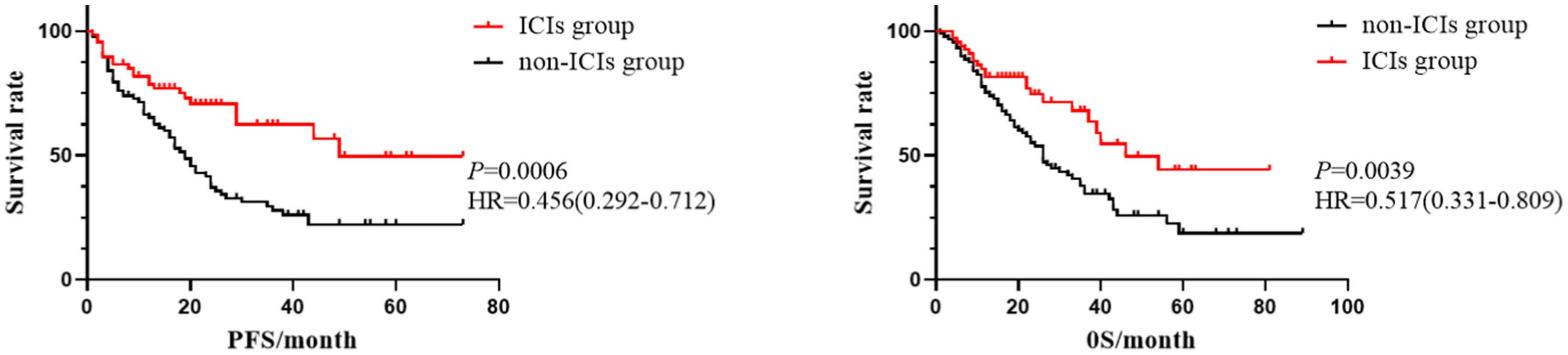
Kaplan–Meier survival curves of PFS and OS in ICIs and non-ICIS groups.

The 155 NSCLC patients were divided into two groups based on their treatment with ICIs: 67 cases(43.2%, 67/155) in the ICI group and 88 cases(56.8%, 88/155) in the non-ICI group. Kaplan–Meier survival analysis curves for PFS and OS were plotted for both groups (Figure 1). The average PFS and OS in the ICI group were 46.55 months and 50.47 months, respectively, compared to 28.60 months and 36.27 months in the non-ICI group. The differences in PFS(*χ^2^* = 11.910, *p* = 0.0006) and OS(*χ^2^* = 8.343, *p* = 0.0039) between the two groups were statistically significant, with patients in the ICI group experiencing longer PFS and OS than those in the non-ICI group.

### 3.3 Univariate and multivariate analysis of ^18^F-FDG PET/CT parameters and clinical features on PFS and OS in the ICIs group

#### 3.3.1 Univariate analysis of ^18^F-FDG PET/CT parameters and clinical features for PFS and OS

All continuous variables in the ICI group were dichotomised based on the ROC curve’s cut-off value. These variables included clinical data such as age, height, weight, BMI, smoking status, Ki-67%, CEA, SCC, NSE, CYFRA21-1, LDH, WBC, NE, ESR, and CRP; PET/CT parameters such as the CT value and maximum diameter of the primary lesion, SUVmax, SUVmean, SUVmin, MTVp, TLGp, TLR, MTVwb, and TLGwb of all lesions; and various categorical variables including gender, smoking history, alcohol consumption, family history of cancer, pathological type, intralobular location of the primary lesion, and the presence or absence of lobulation, burr, cancerous lymphangitis, calcification, pleural effusion, lymph node metastasis, distant metastasis, and stage. These factors were evaluated using the Kaplan–Meier curve for survival analysis and the log-rank test for univariate analysis. The results indicated that factors influencing PFS included smoking history, CT value and maximum diameter of the primary focus, SUVmax, SUVmean, SUVmin, TLR, MTVp, TLGp, MTVwb, and TLGwb(*χ^2^* = 4.224, 5.923, 13.366, 11.036, 4.984, 8.288, 8.906, 26.927, 14.35, 4.86, 13.932, all *p* < 0.05). For OS, the influencing factors were age, smoking history, SCC, primary CT value and maximum diameter, SUVmax, SUVmean, SUVmin, TLR, MTVp, TLGp, and total lesion TLGwb(*χ^2^* = 4.023, 4.931, 4.712, 7.334, 12.497, 9.780, 5.095, 6.705, 7.765, 27.787, 12.487, 12.363, *p* < 0.05) (Table 4).

**Table 4:** Univariate analysis of ^18^F-FDG PET/CT parameters and clinical characteristics for PFS and OS in ICIs group.

PET/CT parameters and clinical characteristics	PFS		OS	
	*χ^2^*	*p*	*χ^2^*	*p*
Age	3.779	0.052	4.023	0.045
Smoking history	4.224	0.040	4.931	0.026
SCC	3.487	0.062	4.712	0.030
CT value	5.923	0.015	7.334	0.007
Maximum diameter	13.366	0.000	12.497	0.000
SUVmax	11.036	0.001	9.780	0.002
SUVmean	4.984	0.026	5.095	0.024
SUVmin	8.288	0.004	6.705	0.010
TLR	8.906	0.003	7.765	0.005
MTVp	26.927	0.000	27.787	0.000
TLGp	14.350	0.000	12.487	0.000
MTVwb	4.860	0.027	3.695	0.055
TLGwb	13.932	0.000	12.363	0.000

#### 3.3.2 Multivariate analysis of ^18^F-FDG PET/CT parameters and clinical features on PFS and OS

**Figure 2:**
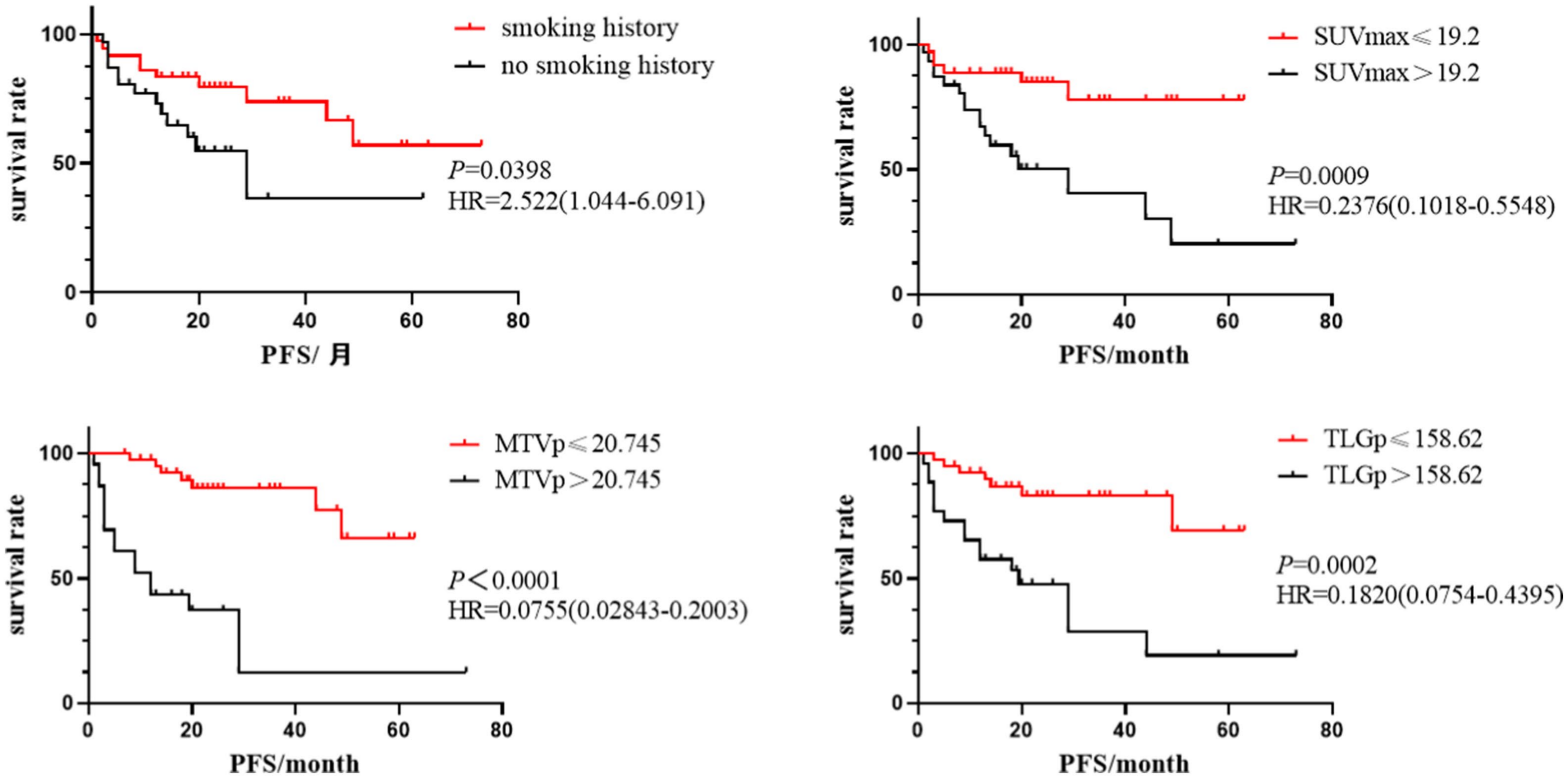
Kaplan–Meier survival analysis curves of smoking history, primary SUVmax, MTVp and TLGp on PFS in ICIs group.

**Figure 3:**
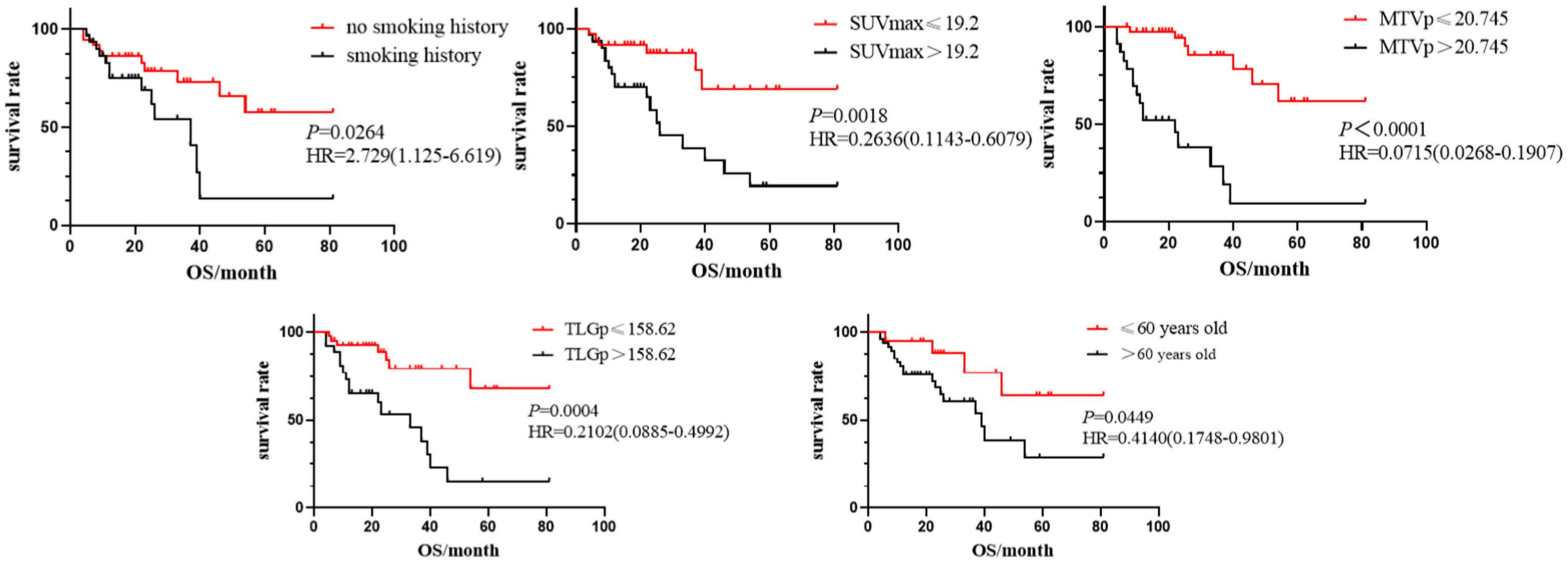
Kaplan–Meier survival analysis curves of smoking history, primary SUVmax, MTVp, TLGp and age on OS in ICIs group.

Statistically significant indicators from univariate analysis were included in Cox multivariate analysis. This analysis identified smoking history[hazard ratio (HR) = 2.522, 95% confidence interval (CI): 1.044 ~ 6.091. *p* = 0.0398], primary SUVmax(HR = 0.2376, 95%CI: 0.1018 ~ 0.5548, *p* = 0.0009), MTVp(HR = 0.0755, 95%CI: 0.0284 ~ 0.2003, *p* < 0.001), and TLGp(HR = 0.1820, 95%CI: 0.0754 ~ 0.4395, *p* = 0.0002) as independent predictors of PFS in the ICI group (Figure 2). These parameters were also independent predictors of OS[HR (95%CI) were 2.729 (1.125–6.619), 0.2636 (0.1143 ~ 0.6079), 0.0715 (0.0268 ~ 0.1907) and 0.2102 (0.0885 ~ 0.4992), respectively, *p* < 0.05), with age(HR = 0.4140, 95%CI: 0.1748–0.9801, *p* = 0.0449) additionally emerging as an independent predictor of OS (Figure 3). The findings indicated that NSCLC patients without a smoking history and with a primary SUVmax ≤19.2, MTVp ≤20.745cm^3^, TLGp ≤158.62 g and age ≤ 60 experienced improved survival benefits following ICI treatment.

**Figure 4:**
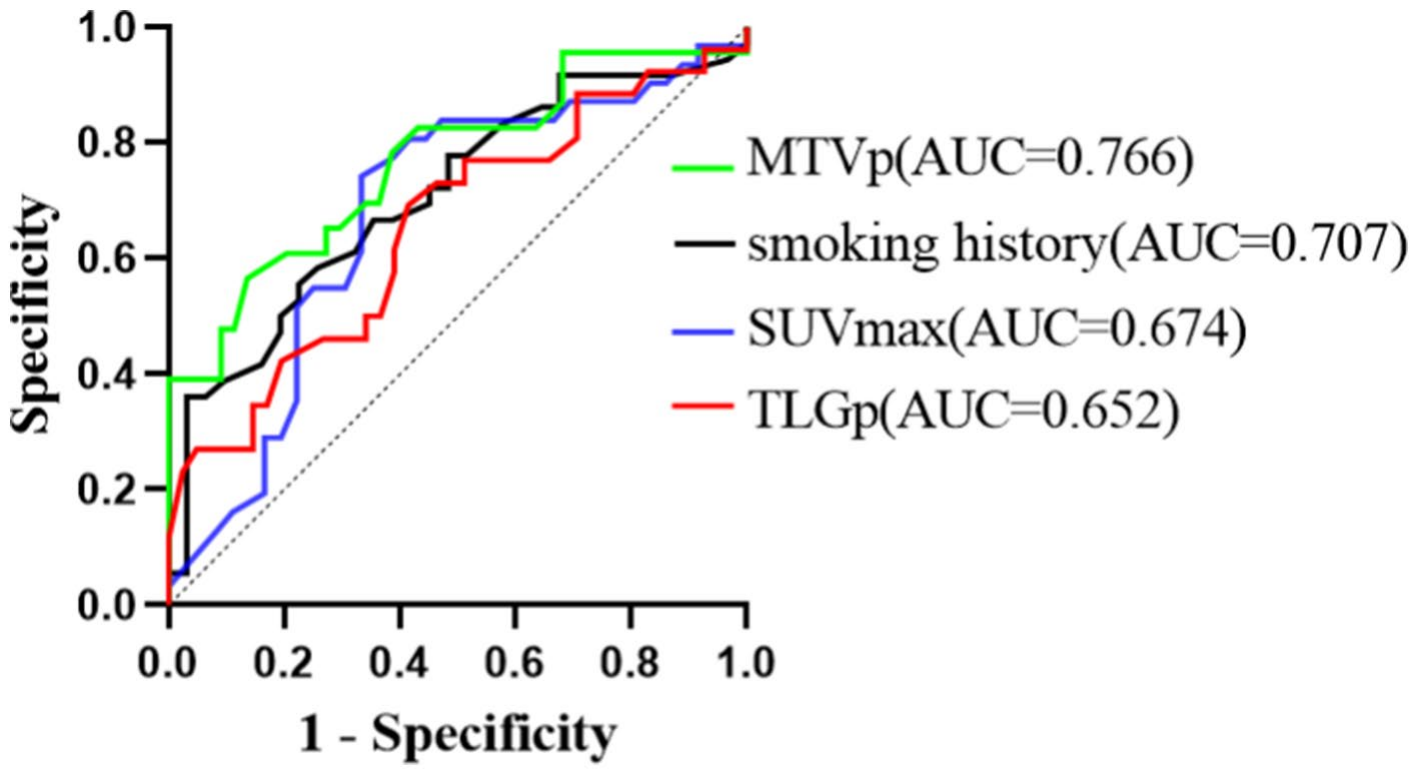
ROC curve of PFS predicted by smoking history, primary SUVmax, MTVp and TLGp in ICIs group.

**Figure 5:**
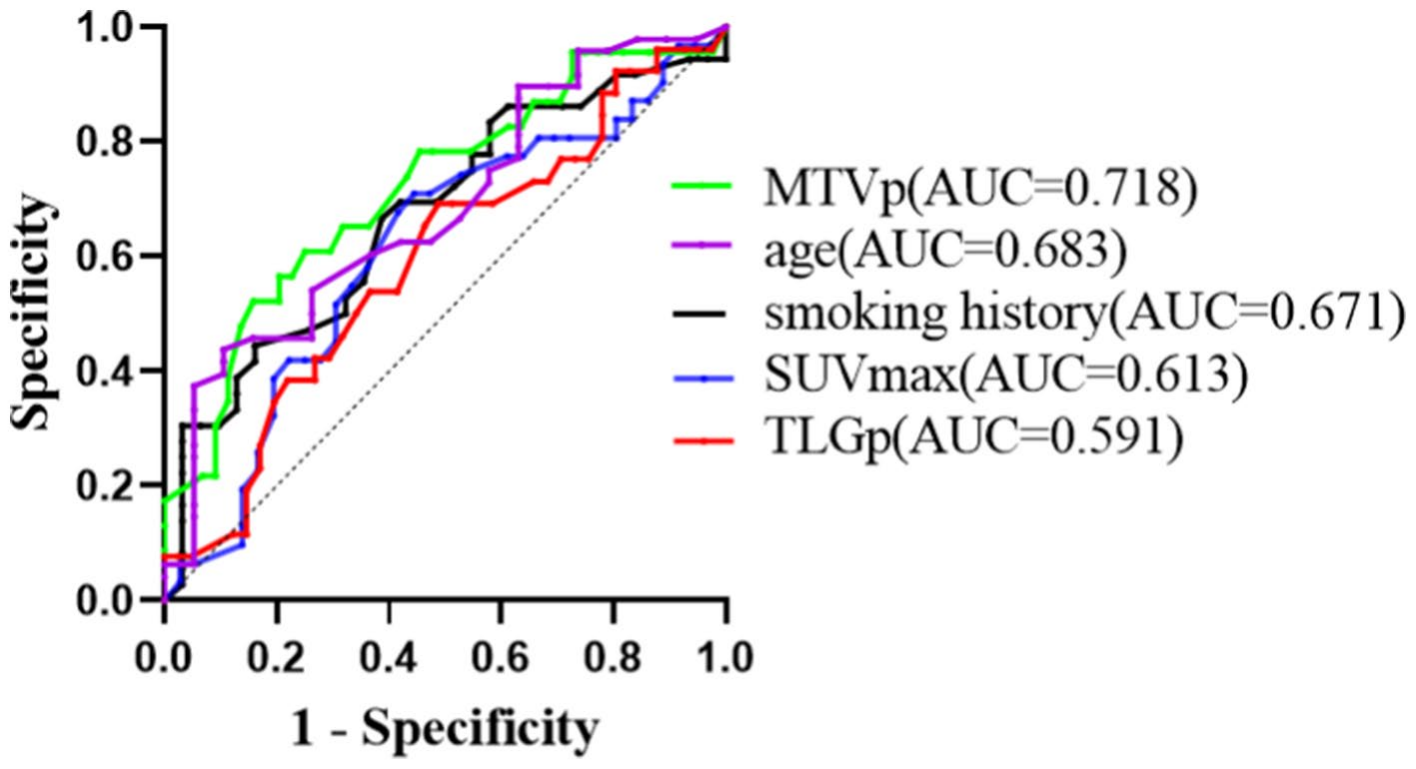
ROC curve of OS predicted by smoking history, primary SUVmax, MTVp, TLGp and age in ICIs group.

**Figure 6:**
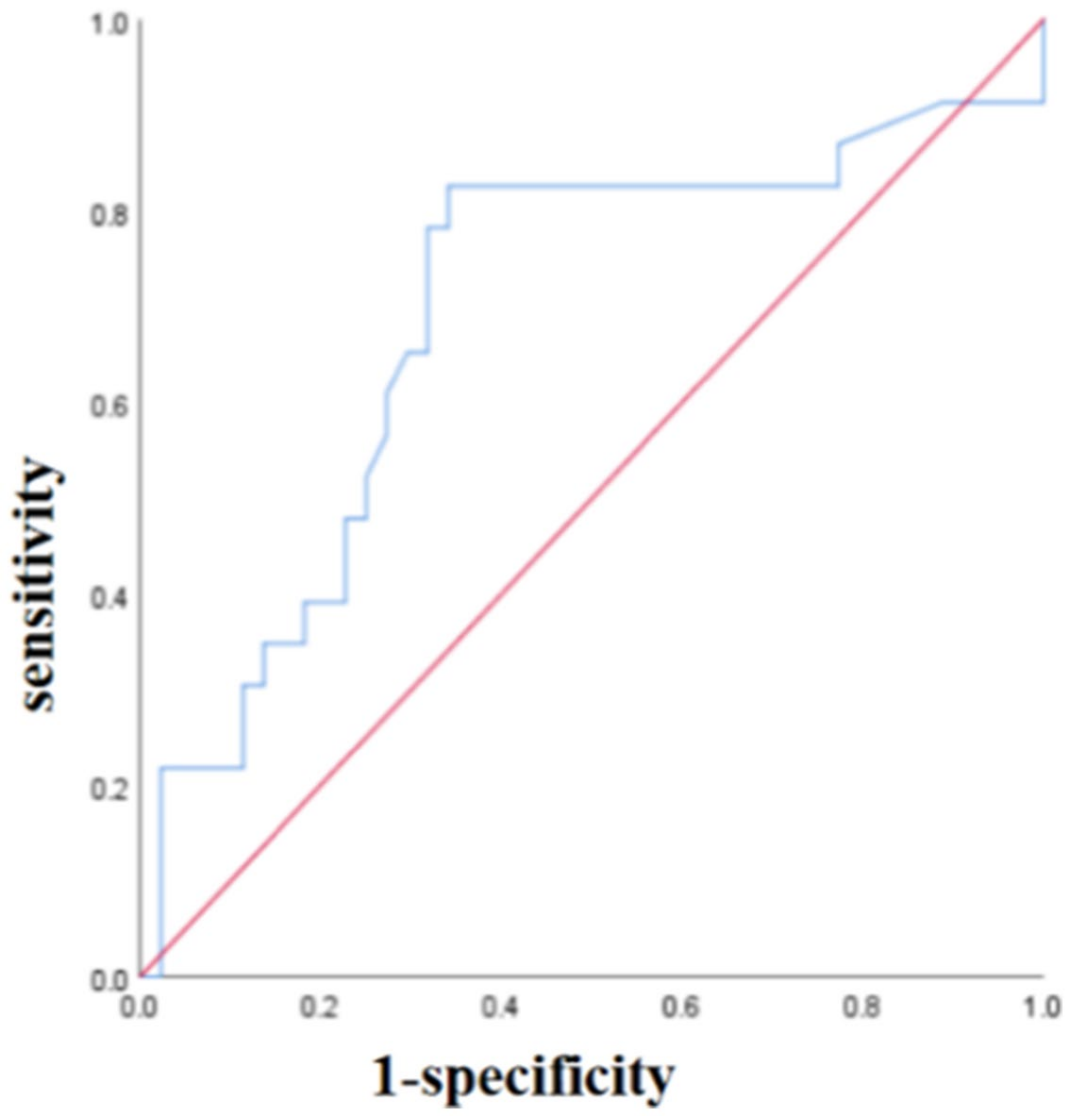
The ROC curve of PFS predicted by smoking history, primary SUVmax, MTVp and TLGp models in ICIs group.

**Figure 7:**
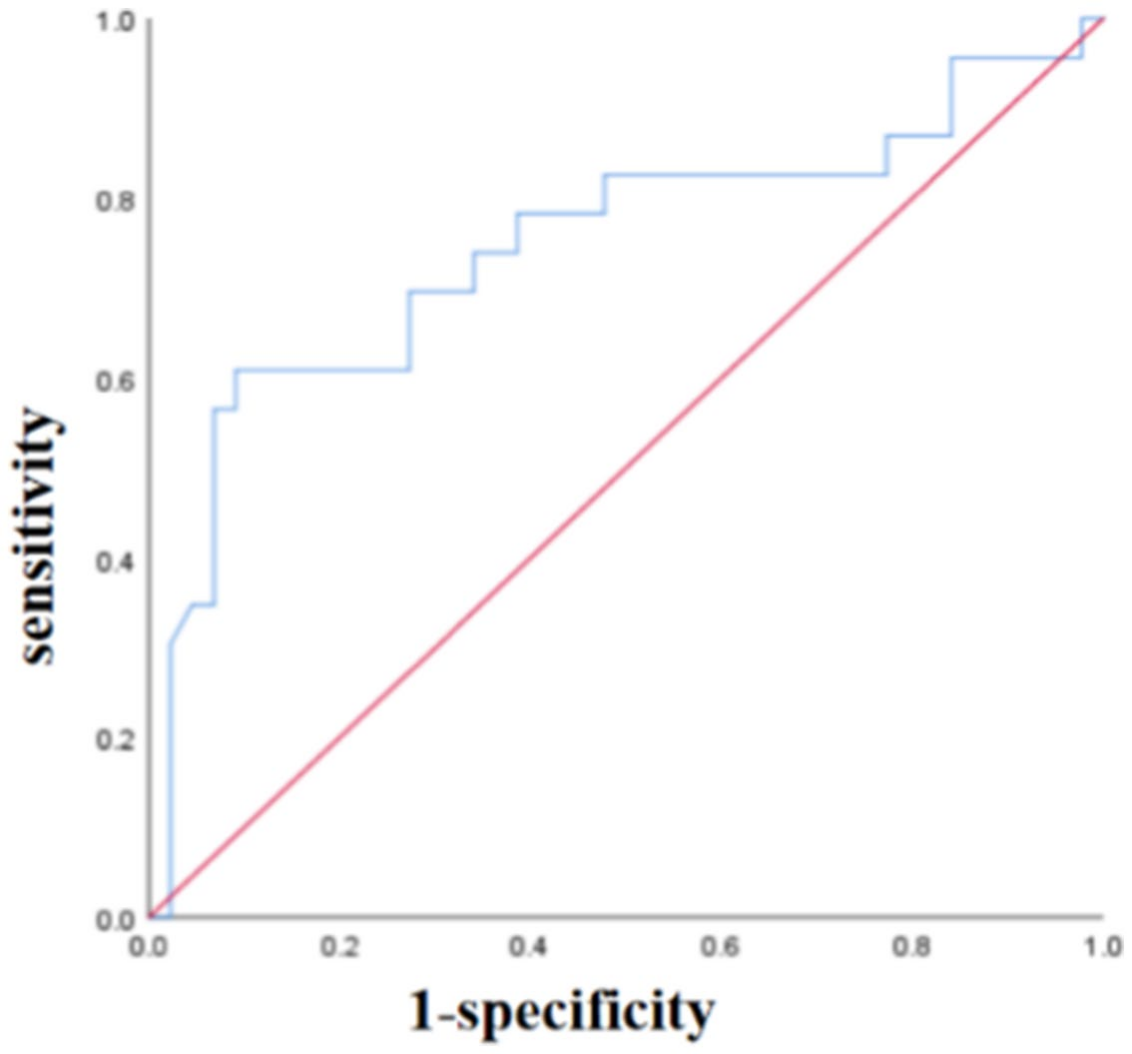
The ROC curve of OS predicted by age, smoking history, primary SUVmax, MTVp and TLGp models in ICIs group.

The sensitivities for predicting PFS were 0.556, 0.742, 0.565, and 0.692 for smoking history, primary SUVmax, MTVp, and TLGp, respectively, with corresponding specificities of 0.774, 0.667, 0.864, and 0.585. The area under curves (AUC) were 0.707, 0.674, 0.766, and 0.652, respectively (Figure 4). For OS, the sensitivities were 0.694, 0.710, 0.522, 0.692, and 0.625 for smoking history, primary SUVmax, MTVp, TLGp, and age, respectively, with specificities of 0.839, 0.556, 0.841, 0.512, and 0.895, and AUC values of 0.671, 0.613, 0.718, 0.591, and 0.683, respectively (Figure 5). A Cox proportional hazards regression model was established to predict PFS and OS in the ICI group, and an ROC curve was used to assess the model’s diagnostic efficiency. The results showed that the sensitivity and specificity of the models based on smoking history, primary SUVmax, MTVp, and TLGp for predicting PFS were 77.3 and 73.9%, with an AUC of 0.692 (Figure 6). For OS, the models based on smoking history, primary SUVmax, MTVp, TLGp, and age had a sensitivity of 65.9%, specificity of 82.6%, and an AUC of 0.748 (Figure 7).

## 4 Discussion

NSCLC constitutes over 80% of all lung cancer cases, with most patients experiencing asymptomatic onset in the early stages. Nearly 70% of newly diagnosed NSCLC patients present at an advanced stage, with a 5-year survival rate of only 10 to 16% (2, 11). Currently, immunotherapy is being applied to some patients with advanced NSCLC. Immune checkpoint molecules are protective elements in the human immune system, normally regulating T cell differentiation and proliferation to maintain immune balance. However, overexpression of these immune checkpoint molecules in tumour tissues inhibits T cell activation and proliferation or induces T cell apoptosis, creating an immunosuppressive tumour microenvironment that allows tumour cells to evade immune surveillance and destruction. The use of ICIs, such as those targeting programmed death 1 (PD-1), programmed death-ligand 1(PD-L1) and cytotoxic T lymphocyte-associated antigen-4 (CTLA-4), blocks the interaction between tumour tissues and T cells, thereby restoring normal immune function and providing significant survival benefits to patients (12). In recent years, tumour immunotherapy has expanded treatment options for patients with advanced NSCLC, with ICIs targeting PD-1, PD-L1, and CTLA-4 becoming a clinical hotspot.

At present, the objective remission rate for NSCLC patients undergoing immunotherapy is only 20%, with notable individual variability and a higher incidence of serious irAEs (6). ^18^F-FDG PET/CT is widely utilised in the diagnosis, efficacy evaluation, and prognosis assessment of lung cancer due to its non-invasive and comprehensive nature globally (13). This study aims to explore the predictive value of ^18^F-FDG PET/CT parameters for ICIs treatment effects in NSCLC patients and to develop a survival prediction model. We retrospectively included 155 NSCLC patients who underwent baseline ^18^F-FDG PET/CT at Hebei General Hospital from January 2016 to April 2023. Our findings suggest that ICIs treatment for advanced NSCLC patients provides significant survival benefits (1, 14). Compared to the non-ICIs group, the PFS and OS were significantly improved in the ICIs group, aligning with previous research results (5). Clinical data analysis revealed that elderly males, with a median age of 66 years, were the most common demographic, and adenocarcinoma was the predominant pathological type, consistent with epidemiological data and earlier studies (15). The baseline ^18^F-FDG PET/CT metabolic parameters in NSCLC patients are closely linked to general data and lesion morphological characteristics. Therefore, these parameters should be assessed in conjunction with other clinical and scientific data to provide a more personalised and accurate diagnosis and treatment plan.

Univariate analysis of ^18^F-FDG PET/CT parameters and clinical characteristics in the ICIs treatment group identified several factors influencing PFS, including smoking history, CT value and maximum diameter, SUVmax, SUVmean, SUVmin, TLR, MTVp, TLGp, MTVwb, and TLGwb. For OS, the influencing factors included age, smoking history, SCC, CT value and maximum diameter, SUVmax, SUVmean, SUVmin, TLR, MTVp, TLGp, and TLGwb. Independent predictors of PFS in the ICIs group were smoking history, primary SUVmax, MTVp, and TLGp. For OS, the independent predictors included age, smoking history, primary SUVmax, MTVp, and TLGp. Smoking history is a well-known risk factor for lung cancer, with studies indicating that the earlier smoking starts, the higher the risk of developing lung cancer, increasing 14 - fold, 8 - fold, and 5 - fold for children, adolescents, and adults, respectively, along with increased mortality (16). In this study, smoking history emerged as an independent predictor of both PFS and OS in NSCLC patients. Xu et al. (17) suggested that smoking status (pack-years), smoking duration, and time to quit smoking could be effective predictors of lung cancer incidence and mortality. However, smoking quantity alone was not an independent risk factor for NSCLC survival in this study, possibly due to the small sample size or inaccuracies in patient smoking data. Ken et al. (5) found that age, primary SUVmax, MTVp, and TLGp were effective predictors of long-term prognosis in NSCLC patients receiving ICIs, aligning with our multifactorial analysis results. Their study also identified carcinomatous lymphangiopathy as an independent predictor, though this finding may vary due to difficulties in diagnosing carcinomatous lymphangiopathy, potentially complicated by other lung conditions like interstitial fibrosis or inflammation. Giulia et al. (15) showed that MTV and TLG are important prognostic factors, positively correlating with disease progression, though SUVmax showed no correlation with PFS and OS, possibly due to differences in study populations and individual disease heterogeneity. In our study, MTVp predicted PFS and OS in ICIs-treated advanced NSCLC patients with AUCs of 0.766 and 0.718, indicating high predictive efficacy. Hye et al. (7) suggested that TLR is an independent prognostic factor for disease recurrence and patient survival in stage IB and IIA NSCLC, though its prognostic value in advanced stages remains unclear and requires further investigation. Karolien et al. (18) found that survival was not related to baseline MTVwb. Similarly, in our study, neither MTVwb nor TLGwb effectively predicted therapeutic response to ICIs in NSCLC patients. The findings indicate that NSCLC patients with no smoking history and primary SUVmax ≤19.2, MTVp ≤20.745cm^3^, TLGp ≤158.62 g and age ≤ 60 who received ICIs treatment experienced better survival benefits. This information could aid in the clinical screening of patients suited for ICIs treatment, improving the objective response and effective response rates and offering NSCLC patients better prognostic outcomes. Based on these results, PFS and OS prediction models for the ICIs group were established using the Cox proportional hazards regression model, with ROC curve analysis showing AUCs of 0.692 and 0.748, respectively, demonstrating good predictive efficiency.

The non-ICIs group did not undergo ICIs treatment for 3 main reasons: firstly, time reasons prevented some patients from applying ICIs treatment. The 155 patients included in this paper were screened from January 2016. Whilst ICIs therapy has emerged in recent years and gradually entered the clinic for the benefit of patients. Secondly, ICIs therapy requires patients to have some clinical indications. Finally, ICIs therapy is currently expensive and requires a certain economic basis to support patients to receive a sufficient course of treatment to achieve the therapeutic purpose. These leads to some patients not being able to apply ICIs.

This study has certain limitations. Although pathological biopsy is crucial for accurately assessing tumour nature and progression, sampling quality can be affected by various factors, and practical issues arise with multiple and continuous pathological examinations. The study did not fully obtain expression data for PD-1 and other immune checkpoints through immunohistochemistry. As a retrospective study, it may be subject to selection bias. Additionally, the absence of an external validation of monocentric data with small sample size and the lack of subgroup analyses for first-line versus second-line ICIs treatment and specific medication regimens limit the generalizability of the findings. The external validation of larger samples of multicenter data is needed in the future.

## 5 Conclusion

(1) Compared with the non-ICIs group, ICIs treatment significantly improves survival and prolongs survival time in NSCLC patients. (2) Smoking history, primary SUVmax, MTVp, and TLGp are independent predictors of PFS in the ICIs group. Age, smoking history, primary SUVmax, MTVp, and TLGp are independent predictors of OS in the ICIs group. All these predictors exhibit good forecasting efficiency. (3) NSCLC patients without a smoking history and with a primary SUVmax ≤19.2, MTVp ≤20.745cm^3^, TLGp ≤158.62 g and age ≤ 60 experience better survival benefits following ICIs treatment.

## Data Availability

The original contributions presented in the study are included in the article/supplementary material, further inquiries can be directed to the corresponding author.
